# Vitamin D3 Metabolites Demonstrate Prognostic Value in *EGFR*-Mutant Lung Adenocarcinoma and Can be Deployed to Oppose Acquired Therapeutic Resistance

**DOI:** 10.3390/cancers12030675

**Published:** 2020-03-13

**Authors:** Tatiana Shaurova, Grace K Dy, Sebastiano Battaglia, Alan Hutson, Letian Zhang, Yunkai Zhang, Christine M Lovly, Mukund Seshadri, David W Goodrich, Candace S Johnson, Pamela A Hershberger

**Affiliations:** 1 Department of Pharmacology and Therapeutics, Roswell Park Comprehensive Cancer Center, Buffalo, NY 14263, USA; tatiana.shaurova@roswellpark.org (T.S.); letian.zhang@roswellpark.org (L.Z.); david.goodrich@roswellpark.org (D.W.G.); candace.johnson@roswellpark.org (C.S.J.); 2 Department of Medicine, Roswell Park Comprehensive Cancer Center, Buffalo, NY 14263, USA; Grace.Dy@roswellpark.org; 3 Center for Immunotherapy, Roswell Park Comprehensive Cancer Center, Buffalo, NY 14263, USA; sebastiano.battaglia@roswellpark.org; 4 Department of Biostatistics and Bioinformatics, Roswell Park Comprehensive Cancer Center, Buffalo, NY 14263, USA; alan.hutson@roswellpark.org; 5 Department of Medicine and Vanderbilt Ingram Cancer Center, Vanderbilt University Medical Center, Nashville, TN 37232, USA; yunkai.zhang@vanderbilt.edu (Y.Z.); christine.lovly@vanderbilt.edu (C.M.L.); 6 Department of Oral Oncology, Roswell Park Comprehensive Cancer Center, Buffalo, NY 14263, USA; mukund.seshadri@roswellpark.org

**Keywords:** lung cancer, EGFR, tyrosine kinase inhibitor, vitamin D, epithelial–mesenchymal transition

## Abstract

EGFR tyrosine kinase inhibitors (EGFR TKIs) are the standard of care treatment for patients with *EGFR*-mutant lung adenocarcinoma (LUAD). Although initially effective, EGFR TKIs are not curative. Disease inevitably relapses due to acquired drug resistance. We hypothesized that vitamin D metabolites could be used with EGFR TKIs to prevent therapeutic failure. To test this idea, we investigated the link between serum 25-hydroxyvitamin D3 (25(OH)D3) and progression-free survival (PFS) in patients with *EGFR*-mutant LUAD that received EGFR TKIs (erlotinib n = 20 and afatinib n = 1). Patients who were 25(OH)D3-sufficient experienced significantly longer benefit from EGFR TKI therapy (mean 14.5 months) than those with 25(OH)D3 insufficiency (mean 10.6 months, *p* = 0.026). In contrast, 25(OH)D3 had no prognostic value in patients with *KRAS*-mutant LUAD that received cytotoxic chemotherapy. To gain mechanistic insights, we tested 1,25-dihydroxyvitamin D3 (1,25(OH)2D3) activity in vitro. 1,25(OH)2D3 promoted epithelial differentiation and restored EGFR TKI sensitivity in models of EGFR TKI resistance that were associated with epithelial–mesenchymal transition (EMT). 1,25(OH)2D3 was ineffective in a non-EMT model of resistance. We conclude that vitamin D sufficiency portends increased PFS among *EGFR*-mutant LUAD patients that receive EGFR TKIs, and that vitamin D signaling maintains drug efficacy in this specific patient subset by opposing EMT.

## 1. Introduction

Lung cancer is the most frequent cause of cancer-related mortality in the US and worldwide [1]. Treatment selection for lung cancer patients is determined by histologic and molecular features of the tumor. Activating mutations in the tyrosine kinase domain of the epidermal growth factor receptor (*EGFR*) gene, including exon 19 deletions (Del19) and exon 21 point mutations (L858R), drive lung tumorigenesis. Such molecular alterations occur in approximately 15% of US and 40% of Asian non-small cell lung cancer (NSCLC) cases [2,3]. Patients with advanced *EGFR*-mutant NSCLC are treated with EGFR tyrosine kinase inhibitors (EGFR TKIs) such as erlotinib (1st generation), afatinib (2nd generation), and osimertinib (3rd generation). In the majority of patients, the clinical course of EGFR TKI anti-tumor activity is characterized by an initial period of efficacy, followed by disease recurrence. There is a critical need to develop strategies to overcome resistance or extend the efficacy of TKIs in this patient population.

Acquired resistance to EGFR TKIs is frequently accompanied by epithelial-to-mesenchymal transition (EMT). This phenomenon is observed not only in preclinical models but also in clinical specimens [4,5,6,7]. Hence, the addition of pro-epithelial agents to the standard treatment of *EGFR*-mutant NSCLC may not only extend the duration of initial response to TKIs but also improve their efficacy after malignant progression. Vitamin D, a secosteroid hormone recognized for its role in muscular–skeletal and immune physiology, is capable of promoting apoptosis and epithelial differentiation in a variety of preclinical tumor models [8,9,10,11,12]. The link between vitamin D status and lung cancer in the clinical setting remains controversial. Several retrospective studies found an association between low serum 25-hydroxyvitamin D3 (25(OH)D3), a commonly used marker of vitamin D status, and increased risk of lung cancer mortality [13,14], while others found no such correlation [15,16,17]. A recently conducted prospective, double-blind placebo-controlled trial of vitamin D supplementation in unselected NSCLC patients demonstrated that supplementation provided no significant survival benefit in the overall cohort [18]. However, the vitamin D deficient subgroup of patients with early-stage LUAD that received vitamin D3 (1200 IU per day) versus placebo had significantly longer progression-free survival (PFS) and overall survival (OS). Notably, this trial was conducted in Japan, where *EGFR* mutations are the predominant oncogenic driver of LUAD. 

In this regard, our previous work demonstrated that *EGFR*-mutant lung tumor models are uniquely sensitive to treatment with the active metabolite of vitamin D, 1,25-dihydroxyvitamin D3 (1,25(OH)2D3). This is largely due to their high expression of vitamin D receptor (VDR) and low levels of a key vitamin D catabolizing enzyme 24-hydroxylase, encoded by the *CYP24A1* gene [19,20]. On the other hand, *KRAS*-mutant NSCLCs appear to have a VDR-low/CYP24A1-high expression pattern and are intrinsically refractory to 1,25(OH)2D3 treatment [19,20]. Cumulatively, these data raise the intriguing possibility that genotype-dependent differences in vitamin D3 signaling capacity exist and that it might be possible to exploit vitamin D3 supplementation preferentially in certain molecular subsets of LUAD to prevent disease progression. The benefits of vitamin D in molecularly defined subtypes of lung cancer have not been previously studied. Driven by the hypothesis that the benefits of vitamin D in patients is likely to be critically influenced by the molecular subtype of LUAD, in the current study we aim to investigate the prognostic value of vitamin D status in *EGFR*-mutant and *KRAS*-mutant NSCLC, as well as its activity in *EGFR* mutant LUAD cells (a candidate responsive subset).

## 2. Results

### 2.1. Serum 25(OH)D3 Predicts PFS in EGFR-Mutant LUAD but Not KRAS-Mutant LUAD

**Figure 1:**
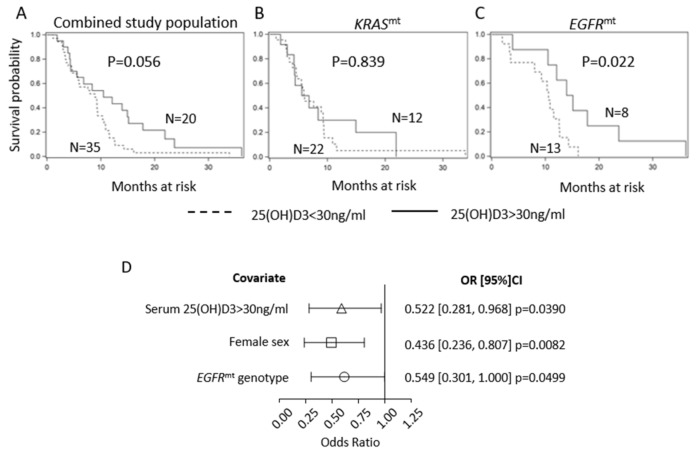
Serum 25(OH)D3 predicts PFS in *EGFR*-mutant LUAD but not *KRAS*-mutant LUAD. Participants were divided into groups based on their serum 25(OH)D3 levels with the cut-off set at 30 ng/mL. Kaplan–Meier progression-free survival (PFS) analyses in combined *EGFR*- and *KRAS*-mutant cohort (**A**), *KRAS*-mutant (**B**), and *EGFR*-mutant (**C**) subsets are shown. (**D**) Forest plot of associations between PFS and serum 25(OH)D3/patient sex/tumor genotype.

To evaluate the association between vitamin D serum concentrations and outcomes in molecularly defined LUAD subtypes, we retrospectively analyzed progression-free survival (PFS) in patients with advanced LUAD, whose tumors harbored either *KRAS* (n = 34) or *EGFR* (n = 21) mutations. Patient characteristics are shown in Table 1 and were balanced across tumor genotypes. All *EGFR*-mutant patients received first-line, standard of care treatment with EGFR TKIs (erlotinib (n = 20) or afatinib (n = 1)). Patients with *KRAS*-mutant LUAD received a platinum-containing regimen as first-line therapy. For survival analysis, participants were divided into groups based on their serum 25(OH)D3 levels, with the cut-off set at 30 ng/mL based on Endocrine Society guidelines [21]. Then, 65% of the *KRAS*-mutant patients (22/34) and 62% of the *EGFR*-mutant patients (13/21) were classified as vitamin D-insufficient using this cut-off. A trend towards longer PFS in the 25(OH)D3 >30 vs. 25(OH)D3 <30 ng/mL groups was detected in the total study population (*KRAS*-mutant and *EGFR*-mutant combined). However, statistical significance was not reached (10.5 months vs. 8.9 months in 25(OH)D3 >30 vs. <30 ng/mL, *p* = 0.056; Figure 1A). We then performed a survival analysis in *KRAS*-mutant and *EGFR*-mutant subsets separately. High serum 25(OH)D3 did not provide PFS benefit in *KRAS*-mutant patients (6.2 vs. 6.0 months in 25(OH)D3 >30 vs. < 30 ng/mL, *p* = 0.839; Figure 1B). Since our previous work determined that vitamin D signaling is compromised in *KRAS*-mutant models of NSCLC, lack of association between 25(OH)D3 and PFS in this subset of patients was not unexpected [19]. Conversely, serum levels of 25(OH)D3 >30 ng/mL predicted significantly longer PFS in the *EGFR*-mutant LUAD patients (14.5 vs. 10.6 months in 25(OH)D3 >30 vs. <30 ng/mL, *p* = 0.022; Figure 1C). At the time of disease progression, when possible, the *EGFR* T790M gatekeeper mutation was screened for as a mechanism of EGFR TKI resistance. The T790M mutation was detected in 38.5% of vitamin D-insufficient and 37.5% of vitamin D-sufficient patients. T790M was tested for but not detected in 53.8% of vitamin D-insufficient and 37.5% of vitamin D-sufficient patients. Material was not available for testing in 7.7% of vitamin D-insufficient and 37.5% of vitamin D-sufficient patients.

Additionally, we found a significant association between PFS and tumor genotype, patient sex, and vitamin D status, where *EGFR* mutation, female sex, and 25(OH)D3 >30 ng/mL directly associated with PFS (Figure 1D). Each of these three factors represented a relative risk reduction in PFS of roughly one half for the reference group relative to the non-reference group. Overall, the above findings suggest that vitamin D may provide survival benefit in a specific, molecularly defined subtype of NSCLC, namely *EGFR*-mutant LUAD. 

### 2.2. EGFR TKI Resistance Is Associated with EMT

One possible explanation for the association between 25(OH)D3 sufficiency and longer benefit from early generation EGFR TKIs is that vitamin D signaling opposes EGFR TKI resistance. To test this idea, we investigated vitamin D receptor-mediated signaling, transcription, and function in models of acquired resistance to EGFR TKIs.

**Figure 2:**
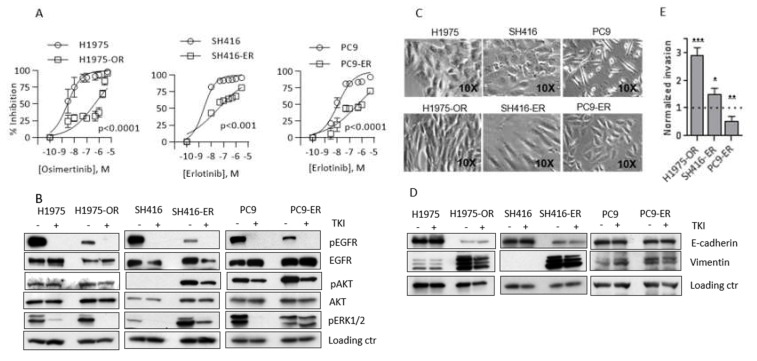
Prolonged exposure to EGFR tyrosine kinase inhibitors (EGFR TKIs) produces cells with different mechanisms of EGFR TKI-resistance. (**A**) H1975 cells were treated with 0.5 µM osimertinib every 72 h for at least 30 days to produce H1975–OR cells. SH416 and PC9 cells were treated with 1 µM erlotinib every 72 h for at least 30 days to produce SH416–ER and PC9–ER cells. Dose-response studies were conducted to determine IC50 and IC80 values for parental cells and their established EGFR TKI-resistant derivatives. Drug response was measured after 9 days of treatment using sulforhodamine B (SRB) assay. The results shown for each model are the average (+/-SD) from 3 independent experiments. (**B**) The indicated cell lines were left untreated (-) or treated (+) with 0.5 µM osimertinib (H1975 and H1975–OR) or 1 µM erlotinib (SH416, SH416–ER, PC9, PC9–ER) for 24 h. Expression of EGFR and its downstream effector molecules were investigated by Western blot. Loading controls used: β-Actin for H1975/H1975–OR, PC9/PC9–ER; α-tubulin for SH416/SH416–ER. Quantitative band analysis is shown in Table S2. (**C**) Images of individual models were captured by phase contrast microscopy. (**D**) Expression of E-cadherin and vimentin was investigated by Western blot as an indicator of EMT. Images are representative of three independent experiments. Loading controls used: β-Actin for H1975/H1975–OR, PC9/PC9–ER; α-tubulin for SH416/SH416–ER. Quantitative band analysis is shown in Table S3. (**E**) Parental cells and their TKI-resistant derivatives were subjected to transwell invasion assay, as described in Methods. The results shown are pooled from 3 independent experiments per cell line. Values obtained for drug-resistant cells were normalized to the corresponding parental cell line (arbitrarily set to an invasion score of 1.0 and represented by the dotted line). * *p* < 0.05; ** *p* < 0.01; *** *p* < 0.005.

*EGFR*-mutant NSCLC cells H1975, SH416, and PC9 were subjected to chronic treatment with 0.5 µM osimertinib (H1975) or 1 µM erlotinib (SH416 and PC9) to generate EGFR TKI-resistant sub-lines, H1975-OR, SH416-ER, and PC9-ER. IC50 and IC80 values for the TKIs were significantly increased in resistant cells as compared to parental counterparts (IC80 values of 14.2 vs. 3010, 11.3 vs. 2248, and 20.9 vs. 27,700 nM for H1975, SH416, and PC9 parental vs. resistant counterparts, respectively; Figure 2A). The emergence of additional mutations in the tyrosine kinase domain of the *EGFR* gene is the most prevalent mechanism of acquired resistance to EGFR TKIs [22,23]. To determine whether EGFR TKI resistance in our models was brought about by second or third site *EGFR* mutations, we sequenced exons 19–21 of the receptor. No additional mutations were found, indicating that the cells developed resistance by an *EGFR*-independent mechanism (Table S1). 

Activation of bypass signaling pathways is another common mechanism of EGFR TKI failure [7,24,25,26,27]. In sensitive cells, EGFR TKIs block phosphorylation of EGFR and its downstream signaling molecules, such as AKT and ERK1/2, resulting in profound cell growth inhibition. However, in cells that acquire bypass-related resistance, these effector cascades remain activated and provide pro-survival signaling even in the presence of EGFR TKIs. To investigate the activation of bypass signaling in our EGFR TKI-resistant models, we examined phosphorylation of AKT and ERK1/2 in the absence and presence of EGFR TKIs. In contrast to SH416 and PC9 parental cells, erlotinib-resistant SH416–ER and PC9–ER cells sustained high levels of either pAKT (SH416–ER) or pERK1/2 (PC9–ER) after treatment with erlotinib. In SH416 parental cells, pAKT was undetected. In SH416–ER cells, pAKT was dramatically upregulated and only modestly reduced by erlotinib, despite complete pEGFR inhibition. Therefore, we conclude that both PC9–ER and SH416–ER cells developed EGFR TKI resistance associated with the activation of a bypass pathway (Figure 2B). Conversely, the effect of osimertinib on pERK1/2 and pAKT was similar in both H1975 parental and H1975–OR cells, suggesting a bypass-independent resistance mechanism (Figure 2B).

Upon microscopic examination, H1975–OR and SH416–ER, but not PC9–-ER cells developed a spindle shape (Figure 2C), suggestive of EMT. Therefore, we examined expression of epithelial marker E-cadherin and mesenchymal marker vimentin in our models. We observed a striking loss of E-cadherin and gain of vimentin in H1975–OR and SH416–ER cells when compared to the corresponding parental lines (Figure 2D). Expression of additional epithelial and mesenchymal markers in H1975–OR cells is shown in Figure S1. These molecular changes were accompanied by a significant increase in invasion in H1975–OR and SH416–ER cells (2.9 and 1.5 times increase in invading fraction in H1975–OR and SH416–ER cells compared to corresponding parental counterparts; Figure 2E). In contrast, expression of E-cadherin and vimentin was similar in PC9 and PC9–ER cells Figure 2D), with PC9–ER cells having a lower invasion potential than their parental counterparts (0.52 relative invading fraction in PC9–ER compared to PC9 parental cells; Figure 2E). Similar results were previously reported by others: PC9 cells with acquired resistance to osimertinib maintained epithelial phenotype, while H1975 osimertinib-resistant cells developed mesenchymal features [28,29,30]. Overall, two out of three EGFR TKI-resistant models we developed (H1975–OR and SH416–ER) underwent EMT. Bypass activation was detected in PC9–ER and SH416–ER cells. Such heterogeneity is not unexpected and is representative of a diverse spectrum of therapy resistance mechanisms in the clinic.

### 2.3. VDR Expression and Signaling Are Maintained in EGFR TKI-Resistant Cells

Vitamin D signaling is initiated when 1,25(OH)2D3 binds to the vitamin D receptor (VDR). Previously, we demonstrated that *EGFR*-mutant treatment-naïve NSCLC cells and tumors express VDR and are sensitive to 1,25(OH)2D3 [19,20]. However, 1,25(OH)2D3 signaling capacity in models of acquired resistance to EGFR TKIs, including those with mesenchymal phenotype, have not been reported. Hence, we sought to characterize the 1,25(OH)2D3 signaling axis in our models of EGFR TKI resistance. In vitro studies were done using the active metabolite of vitamin D, 1,25(OH)2D3. 1,25(OH)2D3 is produced in the kidney and other tissues upon hydroxylation of its circulating precursor, 25(OH)D3, by the *CYP27B1*-encoded, 1α-hydroxylase [31]. Although tumor cells are known to express *CYP27B1* at varying levels (and produce 1,25(OH)2D3), conventionally, in vitro studies of vitamin D activity utilize 1,25(OH)2D3 to avoid variation in local production across models.

**Figure 3:**
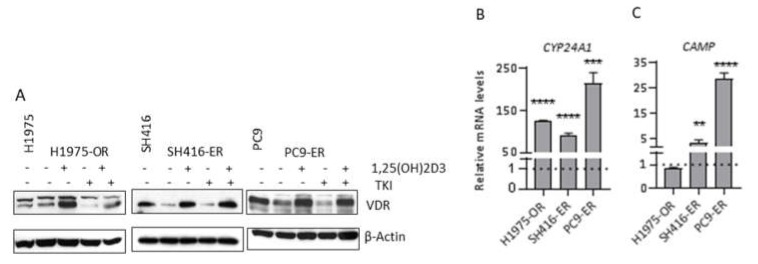
EGFR TKI resistant cells maintain 1,25(OH)2D3 signaling capacity. (**A**) EGFR TKI resistant cells were treated with vehicle control, 100 nM 1,25(OH)2D3, 1 µM erlotinib (SH416–ER and PC9–ER), 0.5 µM osimertinib (H1975–OR), or combination of 1,25(OH)2D3 and the corresponding EGFR TKI for 24 h. (**B**,**C**) EGFR TKI-resistant cells were treated with 100 nM 1,25(OH)2D3 for a total of 96 h and expression of VDR target genes was determined by qRT–PCR. ** *p* < 0.01; *** *p* < 0.001; **** *p* < 0.0001. Western blot analysis was performed at least twice with similar results. Representative images are shown. Quantitative band analysis is shown in Table S4. qRT–PCR was performed using RNA from three independent biological replicates for each cell line. Expression levels in vehicle control-treated cells is set to 1 and shown by the dotted line.

As shown in Figure 3A, VDR protein levels were reduced in SH416–ER and PC9–ER, but not H1975–OR cells when compared to their parental counterparts. Nevertheless, treatment with 1,25(OH)2D3 resulted in expected stabilization and increased levels of the receptor. This effect persisted in the presence of EGFR TKIs in all three resistant cell lines. Furthermore, 1,25(OH)2D3 induced transcriptional responses, as demonstrated by increased mRNA levels of two well-characterized direct targets of VDR, *CYP24A1* and *CAMP*. *CYP24A1* was upregulated in all cell lines under investigation (Figure 3B), and *CAMP* was increased in SH416–ER and PC9–ER (Figure 3C). Therefore, 1,25(OH)2D3 maintains signaling capacity in *EGFR*-mutant, TKI resistant cells, and so may be engaged to combat disease progression.

### 2.4. 1,25(OH)2D3 Promotes Epithelial Differentiation and Cell Cycle Arrest in EMT-Associated Models of EGFR TKI Resistance

We previously reported the ability of 1,25(OH)2D3 to oppose TGFβ-induced EMT in EGFR-mutant, TKI-naïve cells [32]. To investigate 1,25(OH)2D3 activity in EGFR TKI-resistant cells, we performed RNA sequencing of H1975 (Samples 1a, 1b, and 1c), H1975–OR (Samples 2a, 2b, and 2c), and H1975–OR cells treated with 1,25(OH)2D3 (Samples 3a, 3b, 3c). The H1975-based experimental models were selected for RNA sequencing because bypass activation did not contribute to their acquired EGFR TKI resistance. Hierarchical clustering and principal component analyses of the expression data identified a strong deviation from the norm and atypical gene expression pattern in Sample 3a (Figure S2). For these reasons we chose to exclude data obtained from Sample 3a from further analysis.

**Figure 4:**
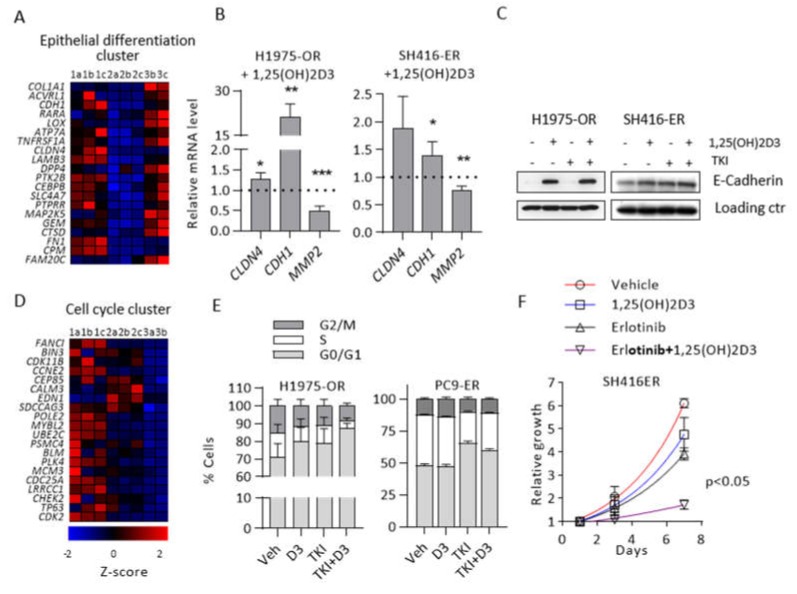
1,25(OH)2D3 promotes epithelial differentiation and cell cycle arrest. RNA sequencing and GSEA were performed in H1975 parental (1a, 1b, 1c) and H1975-OR cells treated with vehicle control (2a, 2b, 2c) or 1,25(OH)2D3 (3b, 3c). (**A**) Top 20 core enrichment genes from the *Epithelial Differentiation* cluster. (**B**). Expression of select epithelial and mesenchymal markers was investigated by qRT–PCR in H1975–OR and SH416–ER cells treated with 1,25(OH)2D3 for 96 h. Expression level in vehicle-treated cells were set to 1 and are represented by the dotted line. * *p* < 0.05; ** *p* < 0.01; *** *p* < 0.001. (**C**) Cells were treated as indicated for 24 h and expression of E-cadherin was determined by immunoblot. Loading controls: β-Actin (H1975–OR) and α-Tubulin (SH416–ER). Quantitative band analysis is shown in Table S5. (**D**) Top 20 core enrichment genes from *Cell Cycle* cluster. (**E**) Cell cycle distribution was analyzed by flow cytometry. Veh, vehicle control; D3, 1,25(OH)2D3; TKI, osimertinib (1975–OR) or erlotinib (PC9–ER). 1,25(OH)2D3 in combination with osimertinib significantly increased fraction of H1975–OR cells in the G0/G1 phase of the cell cycle (adj. *p* < 0.005 vs. vehicle) but not in PC9–ER cells. (**F**) SH416–ER cells were treated as indicated and relative growth was estimated by SRB assay. All experiments were performed in triplicates.

Based on the gene set enrichment analysis (GSEA), an enrichment map was constructed (Figure S3), which revealed that 1,25(OH)2D3 promoted epithelial differentiation in H1975–OR cells. The top 20 genes from the pro-epithelial signature identified by GSEA are shown in Figure 4A. The majority of these core enrichment genes were downregulated in the osimertinib-resistant model compared to the parental cells, while 1,25(OH)2D3 restored their expression in H1975–OR. Targeted qRT–PCR analysis confirmed statistically significant 1,25(OH)2D3-dependent induction of pro-epithelial *CDH1* and *CLDN4*, and downregulation of pro-invasive *MMP2* genes in both H1975–OR and SH416–ER cells (Figure 4B). On the protein level, we detected dramatic upregulation of E-cadherin in H1975–OR cells treated with 1,25(OH)2D3 even in the presence of osimertinib (Figure 4C). 

RNA-sequencing also suggested that 1,25(OH)2D3 promoted cell cycle arrest in H1975–OR cells. The top 20 genes from the cell cycle signature are shown in Figure 4D. It is important to note that the majority of the top 20 cell cycle-related core enrichment genes were significantly downregulated in H1975–OR compared to the parental cells, signifying a prominent slow cycling phenotype associated with EGFR TKI resistance in this model. To validate the effect of 1,25(OH)2D3 on cell cycle distribution, we performed flow cytometric analysis in EGFR TKI resistant cells. 1,25(OH)2D3 in combination with EGFR TKI significantly increased the fraction of H1975–OR cells in G0/G1 phase (71% vs. 87% for vehicle and 1,25(OH)2D3 + TKI, respectively, *p* < 0.005; Figure 4E). Conversely, 1,25(OH)2D3 did not significantly alter cell cycle distribution in EMT-independent PC9–ER cells (Figure 4E). Assaying cell cycle distribution by the means of flow cytometry in SH416–ER cells proved to be technically challenging due to aneuploidy, therefore, we opted to perform SRB-based doubling time analysis in this cell line. Although 1,25(OH)2D3 had little activity as a single agent, it significantly slowed doubling time when combined with erlotinib in SH416–ER cells compared to the vehicle treated cells (2.18 vs. 6.61 days in vehicle and 1,25(OH)2D3 + TKI, respectively, *p* < 0.05; Figure 4F). Detailed statistical analysis of relative cell growth at day 7 in SH416–ER cells is provided in Table S6. The above results provide evidence that 1,25(OH)2D3 promotes epithelial differentiation and increases EGFR TKI-induced cell cycle arrest in *EGFR*-mutant EMT-associated models of acquired resistance to EGFR TKIs.

### 2.5. 1,25(OH)2D3 Promotes Sensitivity to EGFR TKIs in EMT-Associated Models of EGFR TKI Resistance 

**Figure 5:**
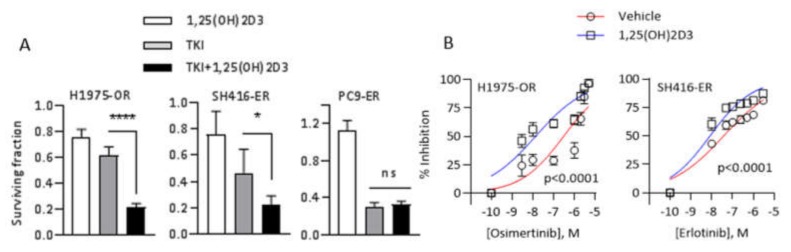
1,25(OH)2D3 restores sensitivity to EGFR TKIs in EMT-associated models of acquired resistance to EGFR targeted therapies. (**A**) H1975, SH416, and PC9 cells and the corresponding TKI-resistant sublines were treated with vehicle control, 1 µM erlotinib (SH416/SH416ER and PC9/PC9–ER) or 0.5 µM osimertinib (H1975/H1975–OR), 100 nM 1,25(OH)2D3, or combination of the above agents every 72 h for a total of 9 days. The number of surviving cells at day 9 in each treatment group was normalized to that in the vehicle control group. * *p* < 0.05; **** *p* < 0.0001. (**B**) H1975–OR and SH416–ER cells were treated with increasing concentrations of the corresponding EGFR TKIs alone or in combination with 1,25(OH)2D3 every 72 h for a total of 9 days. All experiments were performed in triplicates.

Given the ability of 1,25(OH)2D3 to promote epithelial phenotype and deepen EGFR TKI-induced cell cycle arrest in H1975OR and SH416ER cells, we hypothesized that combining 1,25(OH)2D3 with EGFR TKIs in these models would result in improved growth suppression. To test this hypothesis, we first treated H1975–OR, SH416–ER, and PC9–ER cells with either vehicle control, 1,25(OH)2D3, a single concentration of EGFR TKI (0.5 µM osimertinib or 1 µM erlotinib in corresponding cell lines), or the combination of 1,25(OH)2D3 and TKI. As a monotreatment, 1,25(OH)2D3 modestly suppressed the growth of EMT-associated H1975–OR and SH416–ER cells (Figure 5A). The greatest magnitude of growth suppression in the above cell lines was produced by the combination of EGFR TKIs and 1,25(OH)2D3 (79% and 77% growth inhibition in H1975–OR and SH416–ER cells treated with 1,25(OH)2D3 + TKI; Figure 5A). Consistent with the lack of cell cycle suppression observed in Figure 4E, 1,25(OH)2D3 alone or in combination with erlotinib did not impact the growth of PC9–ER cells (Figure 5A). 

Next, we investigated the ability of 1,25(OH)2D3 to improve the efficacy of EGFR TKIs in EMT-associated models of EGFR TKI resistance. H1975–OR and SH416–ER cells were treated with increasing concentrations of osimertinib or erlotinib alone or in combination with 1,25(OH)2D3 and IC50/IC80 values of the TKIs were determined. Addition of 1,25(OH)2D3 significantly reduced concentrations of osimertinib and erlotinib necessary to produce 50% and 80% growth inhibition in both cell lines under investigation (IC80 values of 3010 nM in vehicle-treated vs. 257 nM in 1,25(OH)2D3-treated H1975–OR cells, and 2248 nM in vehicle-treated vs. 218 nM in 1,25(OH)2D3-treated SH416-ER cells; Figure 5B). Additionally, 1,25(OH)2D3 had a similar effect on the efficacy of osimertinib in SH416–OR cells (Figure S4). These cells are osimertinib-resistant derivatives of the SH416 line that have previously been reported to undergo EMT as part of the resistance mechanism [27]. To test whether 1,25(OH)2D3 effects were specific to the drug-resistant state, we examined its interaction with osimertinib in parental H1975 cells. 1,25(OH)2D3 as mono-treatment was growth inhibitory in parental H1975 cells (~20% inhibition, Figure S5A). However, 1,25(OH)2D3 did not increase osimertinib sensitivity in this model (Figure S5B). We note that *EGFR*-mutant parental cells are intrinsically sensitive to EGFR inhibition. Therefore, it may be challenging to demonstrate further improvement in their response to TKIs in vitro. Overall, our results suggest that 1,25(OH)2D3 is capable of improving EGFR TKI efficacy, specifically in the EMT-associated models of acquired resistance to EGFR TKIs.

## 3. Discussion

Previous studies reported conflicting results regarding the link between serum vitamin D and survival in lung cancer patients [13,14,15,16,17,18]. However, the molecular subtypes of tumors have not been taken into consideration. Here, for the first time, we report that vitamin D activity may be restricted to a specific molecular subtype of lung cancer, adenocarcinomas harboring mutations in the *EGFR* gene. We found that high serum vitamin D predicted longer PFS in *EGFR*-mutant, but not in *KRAS*-mutant LUAD patients. In NSCLC, somatic alterations in the *KRAS* gene are reported to occur with at least double the frequency of those occurring in the *EGFR* [33,34]. Hence, in molecularly non-selected cohorts of patients, the survival benefit of vitamin D may not be apparent. Consistent with this interpretation, serum 25(OH)D3 status lost its prognostic value when analyzing our entire study cohort irrespective of genotype. 

Overall, vitamin D insufficiency remains widely prevalent in lung cancer patients. We found that mean serum 25(OH)D3 levels in the total study cohort and in each of the molecularly-defined subsets was below 30 ng/mL in the majority of patients, indicating most patients had insufficient levels of vitamin D based on Endocrine Society guidelines [21]. Several factors may contribute to vitamin D insufficiency, including limited UVB exposure, smoking, and low levels of vitamin D in the diet. We note that some of the patients with *EGFR* mutant LUAD included in our study reported the use of vitamin D supplements. These individuals had higher levels of circulating 25(OH)D3 than those who did not report supplement use [35]. This observation is consistent with a role for variation in vitamin D intake as being determinative for serum 25(OH)D3 levels in patients with *EGFR*-mutant lung cancer. 

Another implication of our data is that vitamin D supplementation may be of critical importance, particularly among lung cancer patients with *EGFR* mutations. We previously demonstrated that *EGFR*-mutant LUADs express significantly lower levels of *CYP24A1* than *KRAS*-mutant cases [19]. *CYP24A1* encodes the 24-hydroxylase that catabolically inactivates 1,25(OH)2D3. Although the mechanistic basis for this relationship remains unknown, variation in tumor 24-hydroxylase levels may contribute to a differential benefit of vitamin D supplementation in patients with *KRAS*-mutant and *EGFR*-mutant tumors. 

It is worth noting that the results of our retrospective study are observational and not without limitations. We recognize that the sample size of the patient cohort is rather small, with only 21 *EGFR*-mutant patients. Despite this, the effect size was sufficiently large to detect a significant correlation. Further, our analysis determined a significant interaction between patient sex, tumor genotype, and PFS. Similar associations were previously reported in larger cohorts of lung cancer patients [36,37,38,39,40]. Additionally, the mean PFS in our *EGFR*-mutant subset of patients was 10.5 months; this, again, is comparable to the duration of response to erlotinib first-line therapy reported earlier [41]. Hence, our study cohort was not atypical compared to larger, previously studied LUAD patient cohorts.

Here, for the first time, we characterize the vitamin D signaling axes in EGFR TKI-resistant models. Previous reports indicate that VDR expression in lung tumors positively correlates with epithelial status [32]. Therefore, vitamin D activity may be compromised in EMT-associated resistance to EGFR TKIs. Here, we demonstrate that EGFR TKI-resistant cells, including those that underwent EMT, maintained VDR expression and are capable of mounting a transcriptional response to 1,25(OH)2D3. In these models, 1,25(OH)2D3 not only enhances the expression of epithelial markers but also induces cell cycle arrest and improves EGFR TKI efficacy. Moreover, we were able to generalize our findings across several cell models and EGFR TKIs. Due to our interest in EMT-associated resistance and the pro-epithelial actions of 1,25(OH)2D3, our current efforts are aimed at developing a clinically relevant orthotopic model of *EGFR*-mutant, TKI-resistant NSCLC. Utilizing such a model will allow us to interrogate not only the anti-growth but also the anti-metastatic potential of 1,25(OH)2D3 in vivo.

1,25(OH)2D3 is known to induce transcription of multiple cell adhesion molecules, including *CDH1* and a number of claudins [42,43]. Moreover, our earlier work showed that concurrent administration of 1,25(OH)2D3 and TGFβ in *EGFR*-mutant TKI-naïve cells opposed TGFβ-driven EMT [32]. In the current study, we uncovered the ability of 1,25(OH)2D3 to support epithelial differentiation in the context of already established EMT. This is an important discovery since it points towards a potential opportunity to utilize vitamin D-based combination therapies in the setting of progressive disease. As a mechanism of resistance to EGFR TKIs, EMT is commonly observed in pre-clinical models [7,27,28,29,30,44]. In the clinic, EMT is believed to cause EGFR TKI failure in a small proportion (~6%) of *EGFR*-mutant LUAD patients [4]. However, the frequency of EMT-associated EGFR TKI resistance in the clinic may be greatly underestimated, as it is not commonly looked for. Intriguingly, in our study, cells that developed resistance to EGFR TKI erlotinib by EMT-independent mechanism (PC9–ER), despite preserved VDR expression and signaling, were refractory to the growth suppressive action of 1,25(OH)2D3. It is possible that the ability of 1,25(OH)2D3 to promote sensitivity to EGFR TKIs is linked to its pro-epithelial activity. On the other hand, according to the catalog of somatic mutations in cancer (COSMIC), PC9 cells harbor mutations in the genes encoding such key regulators of cell cycle progression as CCND2 and CDKN2A [45]. Mutations in these genes might have rendered 1,25(OH)2D3 inactive in this model. Further studies are needed to determine the exact link between EMT and vitamin D activity in EGFR TKI-resistant LUAD.

Another key observation presented here is that vitamin D status was not predictive of PFS in *KRAS*-mutant LUAD patients. As mentioned earlier, *KRAS*-mutant lung cancer cells are refractory to 1,25(OH)2D3 due to perturbations in key determinants of vitamin D signaling, such as low levels of VDR and elevated expression of *CYP24A1* [19]. High local expression of *CYP24A1* is predicted to limit tumor exposure to 1,25(OH)2D3, restricting its efficacy. While this hypothesis offers an explanation of why serum vitamin D levels were not associated with PFS in *KRAS*-mutant patients, it assumes that the major mechanism of action underlying vitamin D’s efficacy is tumor-dependent (as opposed to “host”-dependent). In the retrospective study presented here, *KRAS*-mutation positive patients were treated with cytotoxic chemotherapies, whereas today, such patients may receive immune check point inhibitors. As vitamin D is known to play an important role in the immune physiology, vitamin D adequacy may still be uncovered as a prognostic factor in the context of the “host”-dependent mechanism of its action. In our ongoing investigations, we are looking to delineate the role of tumor *CYP24A1* expression and activity in genotype- and therapy-specific responses to vitamin D. 

In the era of precision medicine, careful selection of patients who may be intrinsically sensitive to the therapeutic agent is critically important. Hence, developing tailored vitamin D supplementation regimens in molecularly defined patient populations is critical to maximizing the utility of vitamin D. Our results suggest that *EGFR*-mutant LUAD patients constitute a population with inherent sensitivity to vitamin D and therefore can derive a benefit from vitamin D-based combination treatments. We propose that further prospective studies aiming to delineate the role of vitamin D in the outcome of NSCLC should focus on the *EGFR*-mutant subset of this malignancy. 

## 4. Materials and Methods

### 4.1. Retrospective Clinical Study

A retrospective, non-interventional biomarker study was conducted to examine the relationship between vitamin D status and progression-free survival in advanced LUAD patients. Analysis of the *KRAS* cohort described below was conducted with the approval of the Roswell Park IRB under non-human subjects research protocol #BDR 117619. Analysis of the *EGFR* cohort was conducted with the approval of the Roswell Park IRB under non-human subjects research protocol #BDR 091817. 

To conduct the study, we first identified a set of patients diagnosed with advanced *EGFR*-mutant LUAD who had a serum sample in the Roswell Park Data Bank and BioRepository Shared Resource (DBBR), were treated with early generation EGFR TKIs (erlotinib n = 20, afatinib n = 1), and for whom outcome data were available. Next, *KRAS* cases were matched to *EGFR* cases based on patient age, sex, disease stage, and race resulting in cohorts with comparable demographics (Table 1). *EGFR* cases were from September 2010 to December 2016, *KRAS* cases were from January 2013 to March 2018. All cases included in the *KRAS* cohort received a regimen containing pemetrexed plus either cisplatin or carboplatin. Patients included in this case series were diagnosed prior to publicly available data showing overall survival benefit of first-line immunotherapy in combination with chemotherapy as a treatment option. *EGFR* and *KRAS* mutations were determined from genomic DNA obtained from tissue specimens subjected to PCR amplification and pyrosequencing to identify sequence variants for specimens tested prior to July 2014 (*EGFR* n = 14, *KRAS* n = 14). All tissue specimen tests from 31 July 2014 onwards were based on next-generation sequencing technology (*EGFR* n = 7, *KRAS* n = 20). *EGFR* variants that were detected are shown in Table S7. De-identified serum samples for the cohort were requested from the DBBR and submitted to Heartland assays for the determination of 25-hydroxyvitamin D3 by gold-standard LC–MS/MS assay. Serum 25D3 levels were linked to annotated clinical information, and the data were submitted to the study statistician for analysis. Progression-free survival (PFS) was determined for each case and defined as time on first line therapy. Patients were classified as vitamin D3-sufficient (≥30 ng/mL 25(OH)D3) or vitamin D3-insufficient (<30 ng/mL) based on Endocrine Society guidelines [21]. In the *EGFR* cohort, two patients, one in each vitamin D arm, underwent EGFR TKI dose reduction. 

### 4.2. Cell Culture

*EGFR*-mutant H1975 cells (*EGFR* T790M, L858R) were purchased from American Type Culture Collection (CRL-5908). *EGFR*-mutant PC9 lung adenocarcinoma cells (*EGFR* Del19) were generously provided by SI Abrams, PhD (Roswell Park Comprehensive Cancer Center, Buffalo, NY, USA). *EGFR*-mutant (*EGFR* Del19) SH416 cells were a gift from CM Lovly, MD, PhD (Vanderbilt University Medical Center, Nashville, TN, USA). H1975 and SH416 cells were cultured in RPMI 1640 (Corning, 1004-CV) supplemented with 10% fetal bovine serum (FBS; Tissue Culture Biologicals 35-010-CV) and 1% penicillin-streptomycin (P/S; Corning, NY, 30-002-CI). PC9 cells were cultured in RPMI 1640 supplemented with 10% FBS, 1% P/S, 7.5% HEPES (Corning, NY, 20-060CI), 1% L-glutamine (Corning, NY, 25-005-CI), 1% sodium pyruvate (Corning, NY, 25-000-CI), and 1% MEM non-essential amino-acids (Corning, NY, 25-025-CI). EGFR TKI-resistant H1975–OR (osimertinib resistant), PC9–ER (erlotinib-resistant), and SH416–ER (erlotinib resistant) sublines were generated by culturing parental cells in growth media containing either 0.5 µM osimertinib (S7297, Selleckchem, Houston, TX, USA) or 1 µM erlotinib (OSI-774-01, OSI Pharmaceuticals, Long Island, New York) for ≥30 days. Concentrations of the TKIs were chosen based on the reported steady state mean serum concentrations of the above drugs at the FDA-approved doses in patients [46,47]. Treatment media was replaced every 72 h. Thereafter, EGFR TKI-resistant cell lines were continuously cultured in growth media containing 0.5 µM osimertinib (H1975–OR) or 1 µM erlotinib (PC9–ER and SH416–ER). Cells were maintained at 37 °C and 5% CO2. All cell lines routinely tested negatively for mycoplasma. *EGFR* mutations in parental cells and their drug resistant derivatives were verified through Sanger Sequencing using the protocol described by Molina-Vila et al. [48] (Table S1).

### 4.3. EGFR TKI and 1,25(OH)2D3 Treatments

1,25(OH)2D3 was kindly provided by Dr. Candace S. Johnson at Roswell Park Comprehensive Cancer Center, Buffalo, NY. For protein and RNA isolation, cells were plated in complete growth media at 40%–50% confluence. The next day, growth media was replaced with treatment media containing 10% charcoal stripped serum (SH30068, HyClone, Pittsburgh, PA) and either vehicle control, EGFR TKI (erlotinib at 1 µM or osimertinib at 0.5 µM), 100 nM 1,25(OH)2D3, or combination of the last two agents at the specified concentrations. For protein analysis, cells were treated for a total of 24 h, with two treatments at time = 0 h (24 h after cell plating, referred to as t0) and at 20 h time points (4 h prior to cell harvest). For RNA analysis, cells were treated for a total of 96 h, with treatments being applied at t0, 48, and 92 h time points. 

### 4.4. Sulforhodamine B (SRB) Assay

Sulforhodamine B (SRB) assay was widely used in this project to estimate cell numbers in a variety of experiments. The utility and methodology of the assay have been described previously [49]. Briefly, cells were fixed with 10% trichloroacetic acid (TCA) (LC262301, LabChem, Zelienople, PA, USA) for 1 h at 4 °C, washed, air dried, and stained with 0.57% (*w/v*) SRB dye (Sigma, S1402) in 1% (*v/v*) acetic acid. The dye was solubilized in 10 mM Tris base and the optical density (OD) at 510 nm was read on BioTek SYNERGY multimode microplate reader. Percent growth inhibition was calculated using the Equation (1):

(1) $$\%\;inhibition=\left(1-\frac{absorbance\;vehicle}{absorbance\;treated}\right)*100\%$$

### 4.5. IC50 and IC80 Determination

Cells were plated in triplicate in complete growth media at 3 × 10^3^ cells per well in 12-well plates and allowed to attach overnight. The next day, cells were treated with increasing concentrations of TKIs (0–5 µM) alone or in combination with 100 nM 1,25(OH)2D3 (as indicated for each experiment). Treatments were reapplied every 72 h for a total of 9 days to capture cytotoxic and cytostatic effects, followed by SRB assay.

### 4.6. Cell Cycle Analysis

Cells were fixed in cold 70% ethanol for a minimum of 30 min. Cells were stained with 0.05 mg/mL propidium iodide (PI, P1304MP, Invitrogen, Waltham, MA, USA) in Krishan buffer (0.1% sodium citrate, JT Baker, 3646-01; 0.02 mg/mL RNAse, Invitrogen, 12091-021; 0.2% Igepal, Sigma I-3021; 1 drop of 1N HCl). Samples were passed through a mesh filter (Corning, NY, #352235), and PI fluorescence was detected by flow cytometry. Cell cycle distribution was determined using ModFit LT 4.0 software (Verity Software House, Topsham, ME, USA). 

### 4.7. Cell Doubling Time Determination

Cells were plated at 3 × 10^3^ cells/well in 12-well plates in triplicate, with a separate plate for each harvesting time point. Cells were harvested every 72 h. Cell growth was determined by SRB assay. Only the exponential growth phase of each curve was considered for cell doubling time calculations. Relative growth is in reference to day one of the exponential growth phase. Doubling times were determined using exponential growth and non-linear regression analysis within GraphPad Prism v7.04 software. 

### 4.8. Transwell Invasion Assay

Cells were plated at 3 × 10^3^ cells per insert in Matrigel covered transwell invasion chambers (354480, Fisher Scientific, Waltham, MA, USA) in 100 µL serum-free media. Then, 700 µL of complete media was placed into the lower chamber. These were designated as invasion wells. Additionally, 3 × 10^3^ cells were plated in 1 mL complete growth media in separate wells of the 24-well plate without inserts (directly into the wells). These were designated as control wells. Then, 48 h after plating, cells were removed from the upper chamber of the invasion wells with a cotton swab. Cells in the control wells and the invasion wells were fixed in 10% TCA and SRB assay performed to determine the cell number. Invasion was calculated using the following formulas:

(2) $$\frac{absorbance\;invasion\;\left(migration\right)\;well}{absorbance\;control\;well}=invading\;fraction$$

(3) $$\frac{invading\;\left(migrated\right)\;fraction\;experimental\;condition}{invading\;\left(migrated\right)\;fraction\;control\;condition}=normalized\;invasion$$

### 4.9. RNA Isolation, cDNA Synthesis, and qRT–PCR

Cells were lysed in TRI-reagent (R2050-1-200, Zymo Research, Irvine, CA, USA) and RNA isolated using Direct-zol RNA miniprep kit (R2052, Zymo Research, Irvine, CA, USA). cDNA was synthesized using High Capacity cDNA Reverse Transcription Kit (Applied Biosystems, 4368814). The SYBR green/ROX/qPCR Master Mix kit (K0221, ThermoFisher Scientific, Waltham, MA, USA) and 7300 Real Time PCR System (Applied Biosystems, Beverly, MA, USA) were used for qRT–PCR reactions. Table S8 contains sequences of the qRT–PCR primers used in the study. Relative mRNA levels were determined using the 2^−ΔΔCt^ method. 

### 4.10. Western Blot Antibodies and Detection Reagents

Cell lysate preparation and Western blotting was performed using the standard lab protocol (20). The following primary antibodies were used for Western blotting: β-Actin (Santa Cruz Biotechnology, sc-1616), Akt (Cell Signaling Technology Danvers, MA, USA (CST), 9272), pAKT(Ser473) (CST, 9271), E-cadherin (BDTransduction Laboratories, San Jose, CA, USA (BD), 610181), EGFR (CST, 4405), pEGFR (Tyr1068) (CS, 2234), pMAPK (ERK1/2) (Thr202/Tyr204) (CST, 9101), α-Tubulin (Millipore, 05-829), vimentin (BD, 550513). The following secondary antibodies were used: Anti-mouse IgG HRP-linked (NA931, GE Healthcare, Chicago IL, USA), anti-rabbit IgG HRP-linked (NA934, GE Healthcare, Chicago IL, USA), anti-rat IgG HRP-linked (NA935V, GE Healthcare, Chicago IL, USA). Bands were detected using Immobilon Crescendo Western HRP substrate (WBLUR0500, Millipore, Burlington, MA, USA). Quantitative band analysis was performed using Image Lab software (Bio Rad, CA, USA). Densitometry values are reported in Table S5, Table S6, Table S7 and Table S8.

### 4.11. RNA Sequencing

RNA was isolated from 3 separate passages of H1975 and H1975–OR cells treated with either vehicle control or 100 nM 1,25(OH)2D3 for 96 h with treatments administered at t0, 48, and 92 h. Sequencing libraries were prepared with the TruSeq Stranded Total RNA kit (20020596, Illumina Inc, San Diego, CA, USA), from 300 ng total RNA according to the manufacturer’s instructions. The DNA libraries were quantitated using KAPA Biosystems qPCR kit and pooled together in an equimolar fashion. Each pool was denatured and diluted to 2.4 pM with 1% PhiX control library (Illumina, FC110-3001) added. The resulting pools were loaded into the 150 cycle NextSeq high output Reagent cartridge (FC-404-2002, Illumina Inc, San Diego, CA, USA) for 75-cycle paired-end sequencing and sequenced on a NextSeq500 following the manufacturer’s recommended protocol (Illumina Inc, San Diego, CA, USA). Data are available via the Gene Expression Omnibus (GEO) repository, accession GSE146850.

### 4.12. Bioinformatics

Raw FASTQ files were mapped onto the hg38 human genome using the Kallisto RNAseq quantification program [50], and gene level counts were obtained, summarizing transcript information with the TX import R package [51]. Gene set enrichment and leading edge analyses were performed using the Broad Institute GSEA 3.0 application [52]. RNA sequencing expression data were compared to the Gene Ontology (GO) dataset from molecular signature databases (MSigDB v6.2) [53]. An enrichment map was constructed using Cytoscape v3.6.1 software and the Enrichment map plugin [54]. Cytoscape Autoannotate application was used for gene set clustering, followed by manual curation of the clusters and annotations based on the GO categories (Figure S3). 

### 4.13. Statistical Methods

Patients’ baseline characteristics (age, stage, and sex) and biomarker values in relation to progression-free survival were compared, and Cox proportional hazards regressions were used to evaluate associations in terms of the corresponding hazard ratios and 95% confidence intervals. Graphical estimates of survival probabilities relative to progression-free survival were estimated using Kaplan–Meier estimators. All statistical tests were two-sided, and a *p*-value of <0.05 was considered statistically significant. All analyses were performed using SAS 9.4 (SAS Institute, Cary, NC, USA). The sample size was dictated by sample availability and was fixed at n = 55.

Unless specified otherwise, in vitro experiments were performed in three biological replicates. Data are presented as mean ±SD. Statistical analyses were performed in GraphPad Prism v7.04 software. Dose-response and doubling time studies were analyzed using non-linear regression. Non-linear models were fit to dose-response data and the LogIC50 and LogIC80 values were compared between the groups using an F-test. For relative mRNA expression and invasion assays, statistical significance was determined by two-sample *t*-tests. For the cell cycle distribution, statistical significance was calculated using 2-way ANOVA followed by Tukey’s multiple comparison test. A *p*-value of <0.05 was considered statistically significant.

## 5. Conclusions

Vitamin D insufficiency is common among lung cancer patients. Although vitamin D has been shown to exert anti-tumor activity in multiple pre-clinical tumor models, its role in advanced lung cancer remains controversial. Here, for the first time, we investigated the prognostic value of vitamin D sufficiency in genotype-specific cohorts of advanced LUAD. Our results indicate that vitamin D has LUAD subtype-selective effects on clinical outcomes: *EGFR*-mutant patients derive a survival benefit from higher vitamin D levels, while *KRAS*-mutant patients do not. Mechanistically, vitamin D signaling promotes the expression of epithelial markers in *EGFR*-mutant cells and improves the efficacy of EGFR TKI treatment in vitro. Hence, vitamin D supplementation may be a safe and cost-effective tailored treatment approach for improving clinical outcomes in *EGFR*-mutant LUAD patients.

## Figures and Tables

**Table 1:** Patients’ characteristics. Tumor stage and patients’ demographic characteristics were balanced across tumor genotypes.

Characteristic	*EGFR*^mt^ (n = 21)	*KRAS*^mt^ (n = 34)
Age (y), mean (SD)	68 (9)	65 (9)
Female, No. (%)	14 (64)	19 (56)
African American, No. (%)	2 (10)	3 (9)
Stage, No. (%)		
IV	21 (100)	34 (100)
Serum 25(OH)D3 (ng/ml), mean (SD)	28.78 (11.13)	24.84 (12.27)

## Supplementary Materials

The following are available online at https://www.mdpi.com/2072-6694/12/3/675/s1. Table S1. EGFR genotypes for the models used in the current study. Table S2. Western blot densitometry analysis related to Figure 2B. Table S3. Western blot densitometry analysis related to Figure 2D. Table S4. Western blot densitometry analysis related to Figure 3A. Table S5. Western blot densitometry analysis related to Figure 4C. Table S6. Statistical analysis of SH416-ER relative cell growth at day 7. Table S7. EGFR variants detected prior to therapy initiation in the EGFR-mutant cohort. Table S8. qRT-PCR primers and sequences. Figure S1. H1975-OR cells undergo EMT. Expression of epithelial (blue) and mesenchymal (red) markers was determined by qRT-PCR analysis. mRNA levels were normalized to those found in the parental H1975 cells (set to 1 and shown as the dotted line). **, p < 0.01; ***, p < 0.001; ****, p < 0.0001; #, p < 0.00001. N = 3. Figure S2. Quality assessment analysis of the RNA sequencing results identified 3a sample as a deviation from the norm. Hierarchical clustering (A) and principal component analysis (B) revealed 3a as an atypical sample that strongly deviated in gene expression pattern from other samples in the same treatment group, as well as other samples in the same cell line. Therefore, sample 3a was excluded from further gene set enrichment analysis. Figure S3. Enrichment map of upregulated (red) and downregulated (blue) GO categories in H1975-OR cells treated with 1,25(OH)2D3. Significantly enriched gene sets are represented by colored dots. Gene sets are grouped into clusters (circles) based on the description of ontological categories to illustrate up- and down-regulated biological processes in H1975-OR cells treated with 1,25(OH)2D3. Figure S4. SH416-OR cells were treated with increasing concentrations of osimertinib with or without 1,25(OH)2D3 every 72h for a total of 9 days. Percent growth inhibition was determined by SRB assay. Significant decrease in osimertinib IC50 was found in the cells treated with 1,25(OH)2D3 (IC50 = 33.3 nM) compared to those treated without (IC50 = 217 nM). N = 3. Figure S5. 1,25(OH)2D3 has no effect on EGFR TKI-sensitivity in H1975 parental cells. A. H1975 cells were treated as indicated every 72 h for a total of 9 days. Surviving cell fraction relative to vehicle control treated cells was determined by SRB assay. ****, *p* < 0.0001. B. H1975 cells were treated with increasing concentrations of osimertinib with or without 100 nM 1,25(OH)2D3 every 72 h for a total of 9 days. Percent growth inhibition was determined by SRB assay.
