# Supplementary material for: Vitamin D3 Metabolites Demonstrate Prognostic Value in EGFR-Mutant Lung Adenocarcinoma and Can be Deployed to Oppose Acquired Therapeutic Resistance
Source: Cancers (Basel). 2020 Mar 13;12(3):675. doi: 10.3390/cancers12030675 (PMC7140110; doi:10.3390/cancers12030675)
Supplement: Supplementary file 1 [file cancers-12-00675-s001.zip › 717869-supplemental file-final/cancers-717869 - western blot figures.pdf]

Related to Figure 2B; H1975 and H1975-OR cells

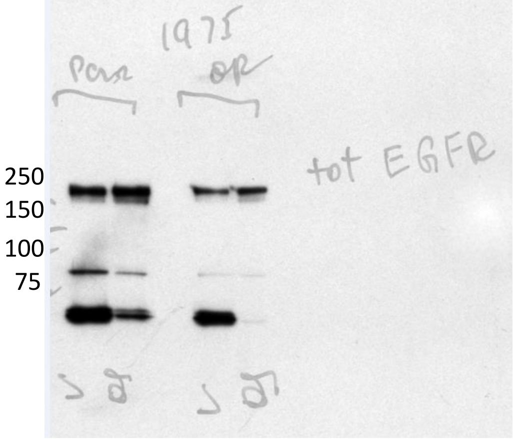

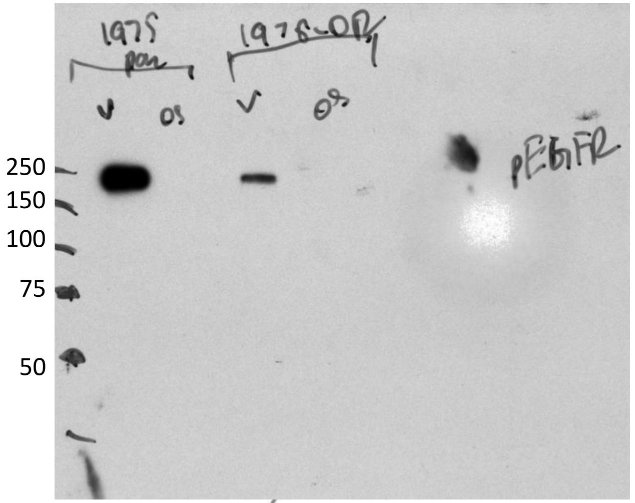

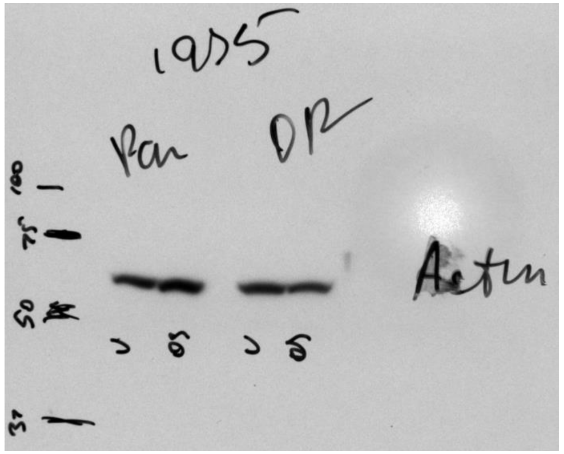

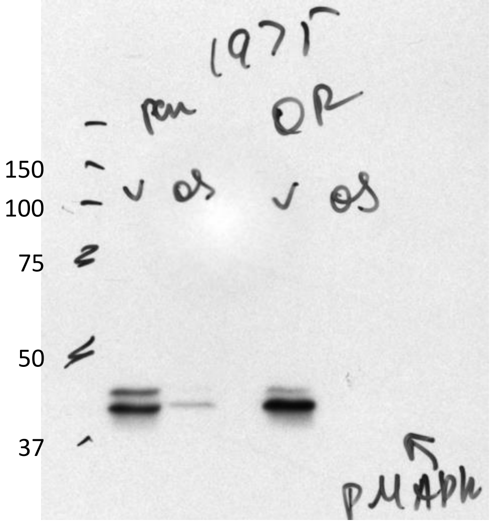

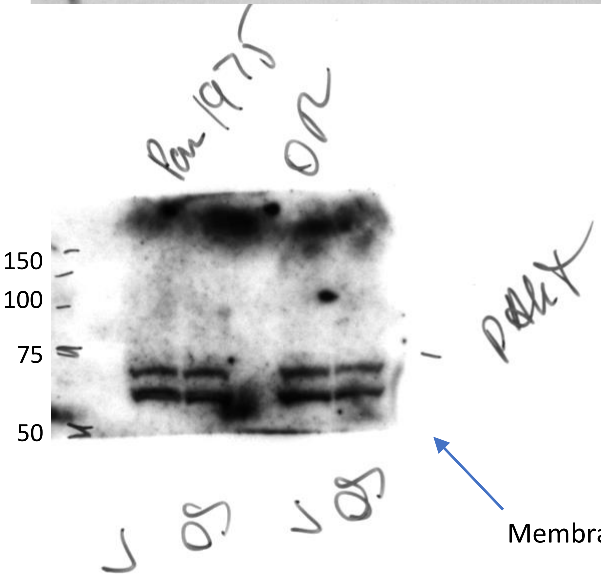

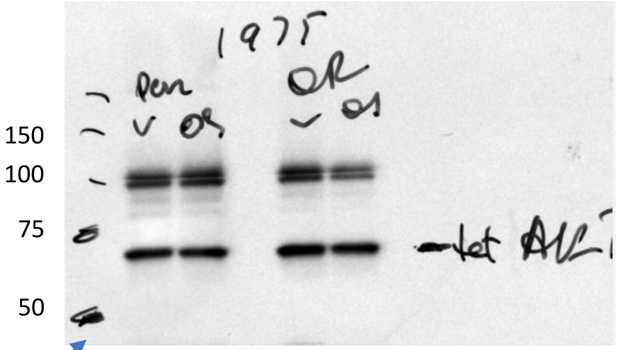

Membrane cut

Membrane was serially probed. Bands at ~100kDA appearing On the total AKT blot are from prior STAT3 detection (not discussed in the current manuscript).

Protein	H1975 veh	H1975 osim	H1975-OR veh	H1975-OR osim
pEGFR	1	0	0.50162867	0
EGFR	1	0.826981	0.515671	0.598515
pAKT	1	1.089721	0.839346	0.769347
AKT	1	0.798991	1.218423	1.244072
pERK1/2	1	0.091692	0.935976	0.025666

Related to Figure 2B; SH416 and SH416-ER cells

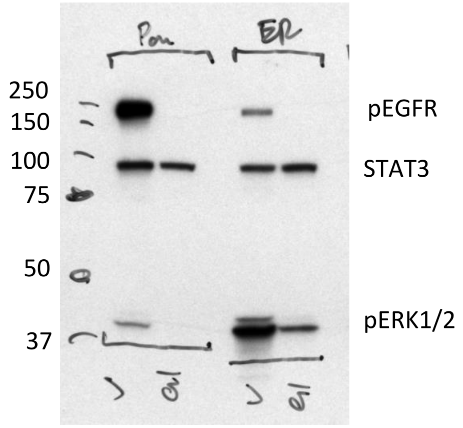

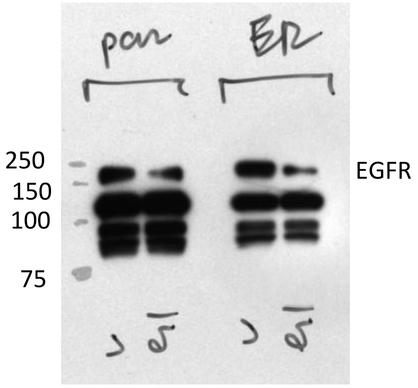

Membrane was serially probed. Bands at ~100kDa are from prior detection of STAT3 (not discussed in this manuscript). Bands of ~40kDa appearing on pAKT and AKT images are from previous pERK1/2 detection.

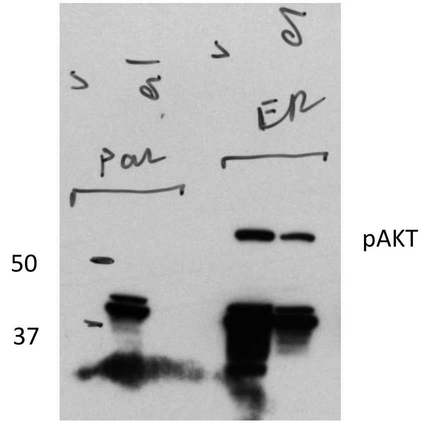

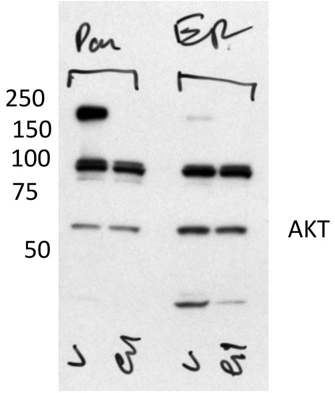

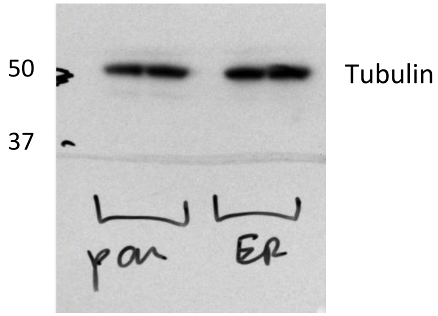

Protein	SH416 veh	SH416 erlo	SH416-ER veh	SH416-ER erlo
pEGFR	1	0	0.254172271	0
EGFR	1	0.645992	1.067296	0.485255
pAKT	0	0	1	0.705949963
AKT	1	0.908377	1.514607	1.35811
pERK1/2	1	0.384064	3.196798	1.649059

Related to Figure 2B; PC9 and PC9-ER cells

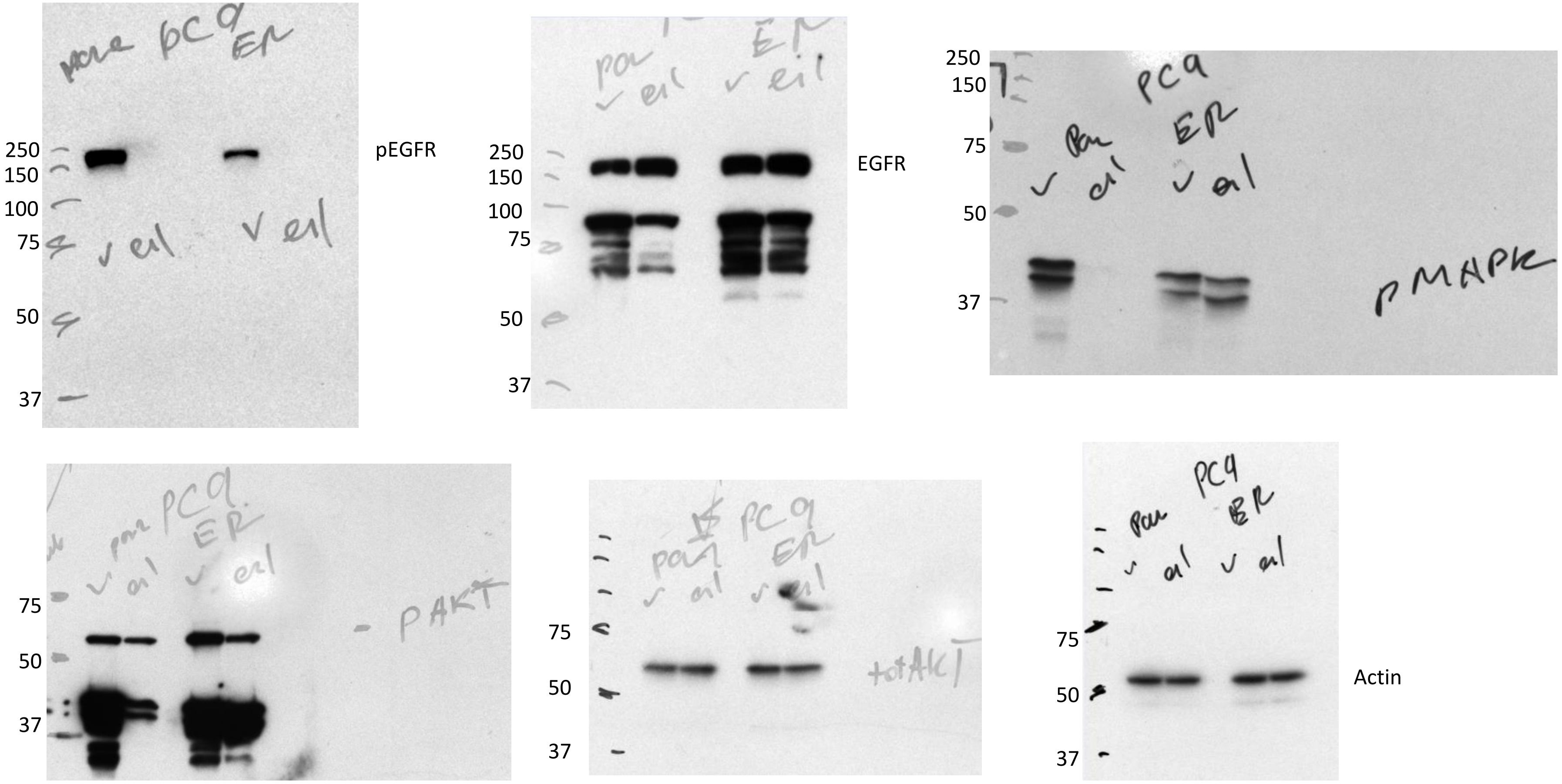

	PC9 veh	PC9 erlo	PC9-ER veh	PC9-ER erlo
pEGFR	1	0	0.209609814	0
EGFR	1	1.371217	1.327668	1.50625
pAKT	1	0.509423	1.221005	0.814961
AKT	1	1.366308	1.256457	1.052674
pERK1/2	1	0.037584	0.574764	0.640738

Membrane was serially probed. Bands of ~40kDa appearing on the pAKT image are from prior pERK1/2 detection. Bands of ~90kDA appearing on the EGFR blot are from prior detection of EZH2 (not discussed in this manuscript)

Related to Figure 2D; H1975 and H1975-OR cells

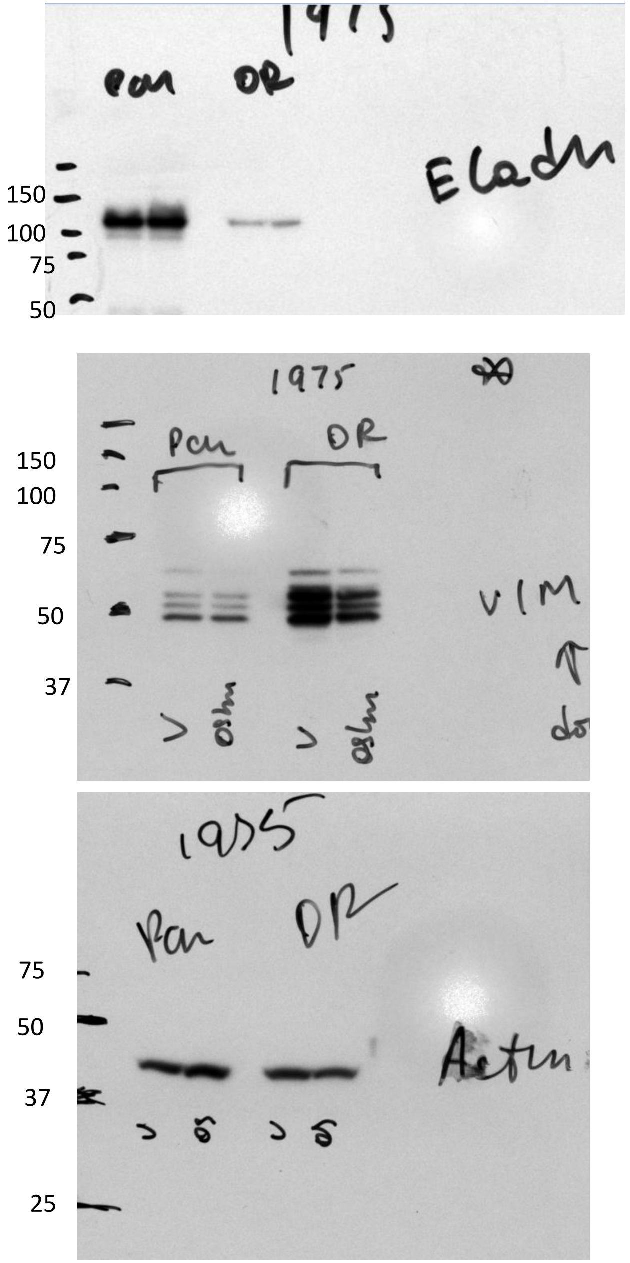

Protein	H1975 veh	H1975 osim	H1975-OR veh	H1975-OR osim
E-cadherin	1	0.864641	0.216314	0.243656
Vimentin	1	0.990695	3.208353	2.923499

Related to Figure 2D; SH416 and SH416 ER cells

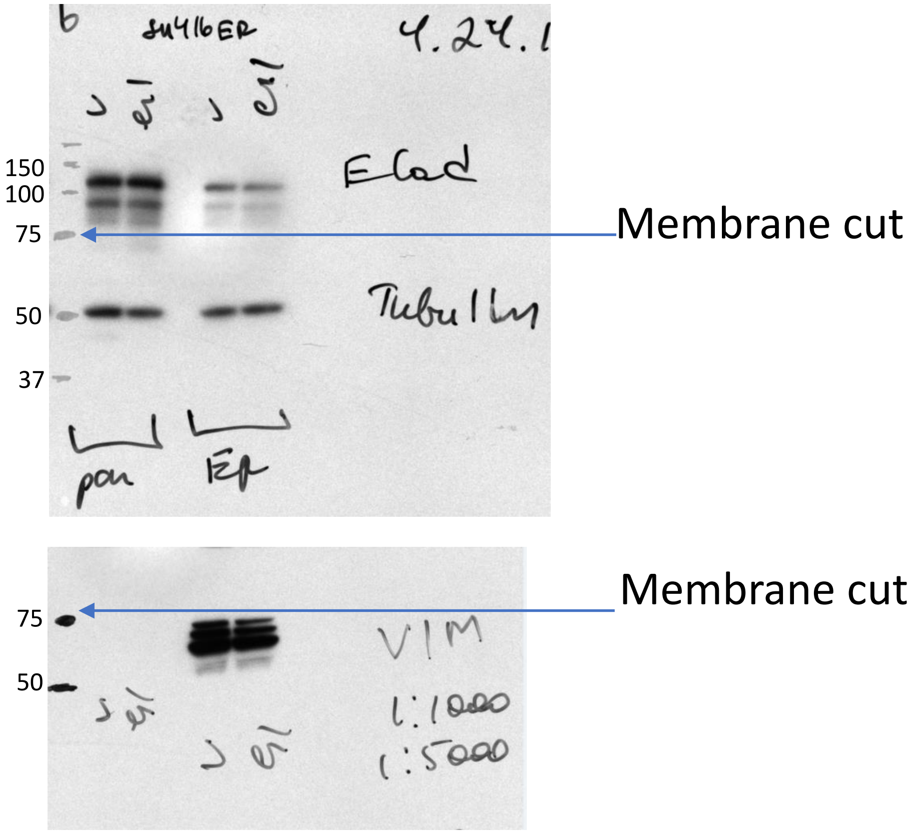

Protein	SH416 veh	SH416 erlo	SH416-ER veh	SH416-ER erlo
E-cadherin	1	1.271376	0.585481	0.493888
Vimentin	0	0	1	0.944333

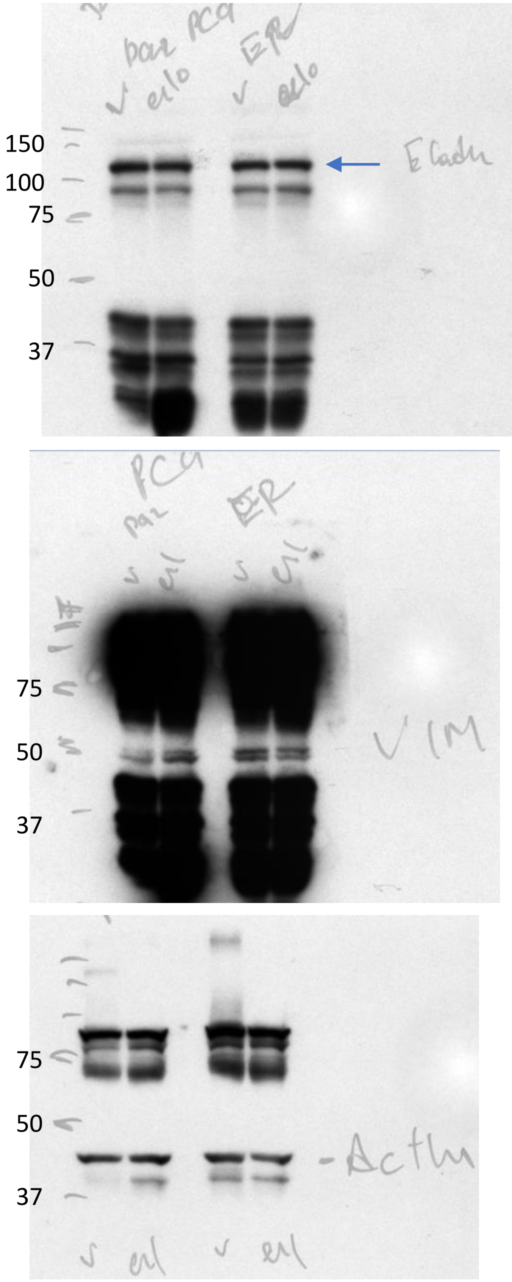

Related to Figure 2D; PC9 and PC9-ER cells

Protein	PC9 veh	PC9 erlo	PC9-ER veh	PC9-ER erlo
E-cadherin	1	0.760619	0.862854	0.976294
Vimentin	1	1.242039	1.326406	1.145299

Membrane was serially probed. Strong background bands on the vimentin blot are from previous E-cadherin detection.

Related to Figure 3A; H1975-OR and PC9-ER cells

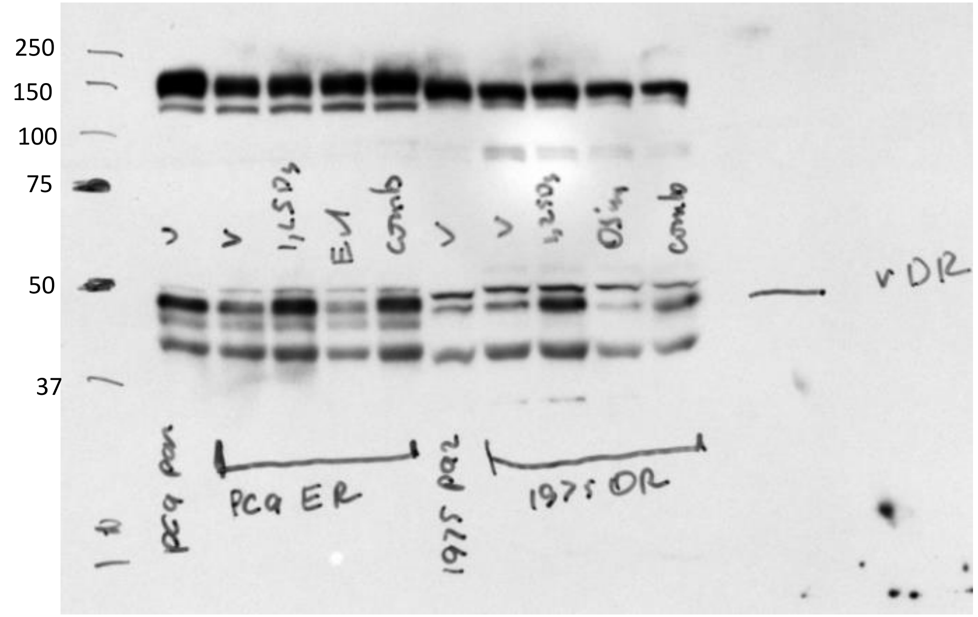

Protein	H1975	H1975-OR veh	H1975-OR 1,25(OH)2D3	H1975-OR osim	H1975-OR combo
VDR	1	0.917038	2.02483	0.521215	1.582157

Protein	PC9	PC9-ER veh	PC9-ER 1,25(OH)2D3	PC9-ER erlo	PC9-ER combo
VDR	1	0.83627	1.172567	0.594546	1.07031

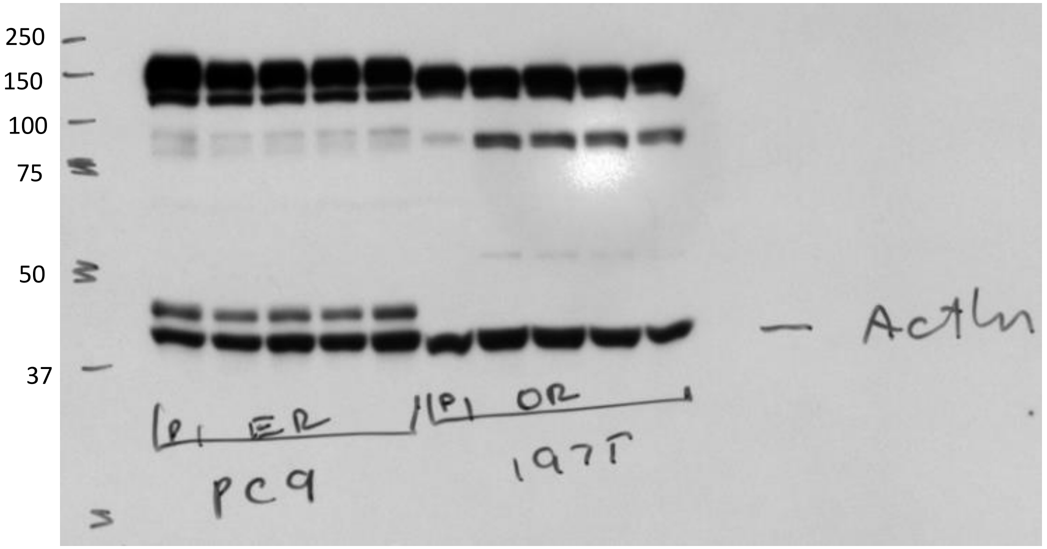

Membrane was serially probed. Bands appearing on both images between 75-150 kDa are from prior detection of AXL (not discussed in the current manuscript).

Related to Figure 3A; SH416-ER cells

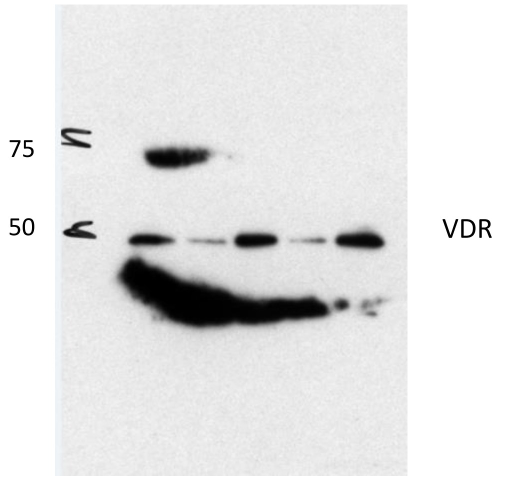

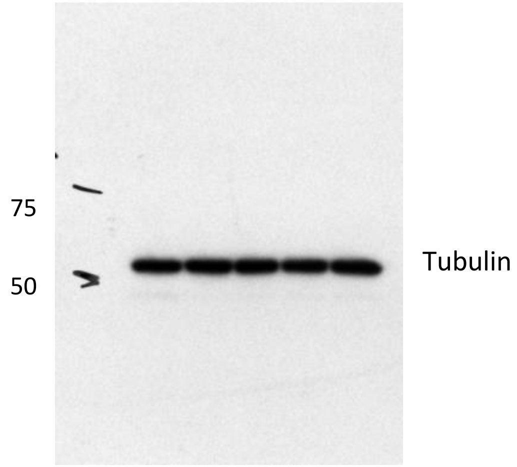

Protein	SH416	SH416-ER veh	SH416-ER 1,25(OH)2D3	SH416-ER erlo	SH416-ER combo
VDR	1	0.231282	1.289263	0.206617	1.321116

Related to Figure 4C; H1975-OR cells

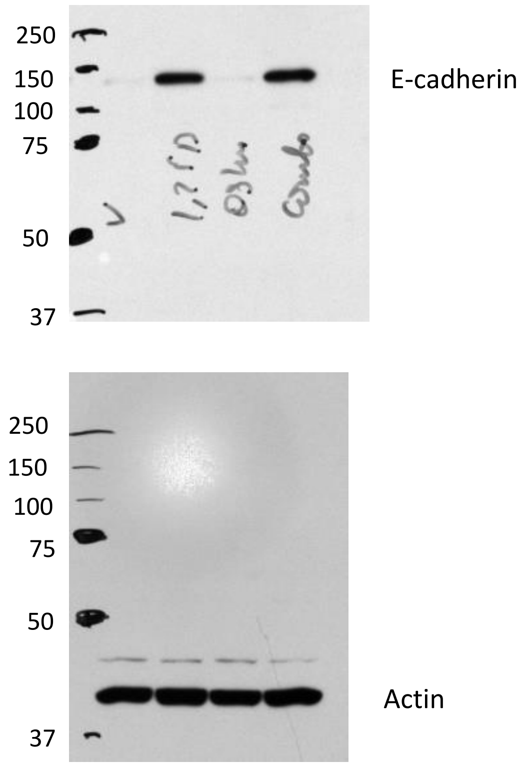

Related to Figure 4C; SH416-ER cells

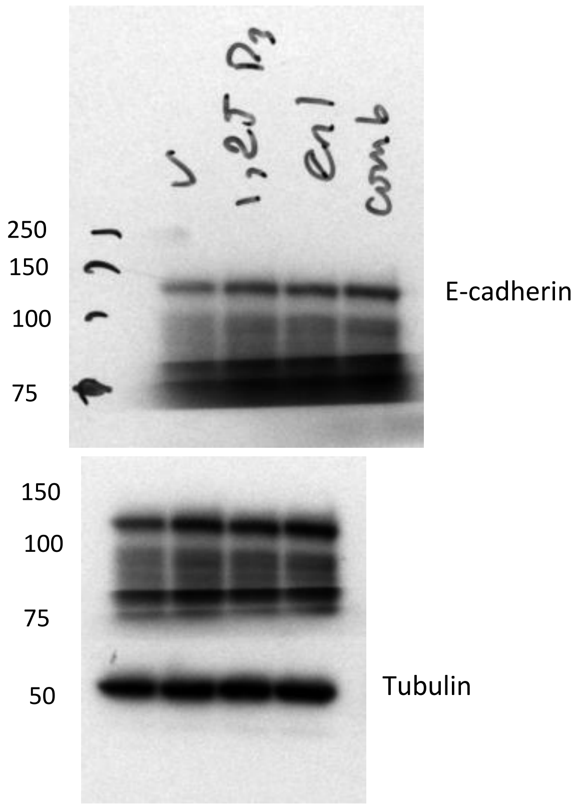

Protein	H1975-OR veh	H1975-OR 1,25(OH)2D3	H1975-OR osim	H1975-OR combo
E-cadherin	1	27.02011	0.768365	29.08314

Protein	SH416-ER veh	SH416-ER 1,25(OH)2D3	SH416-ER osim	SH416-ER combo
E-cadherin	1	1.22253	1.084709	1.199782
