# Supplementary material for: Vitamin D3 Metabolites Demonstrate Prognostic Value in EGFR-Mutant Lung Adenocarcinoma and Can be Deployed to Oppose Acquired Therapeutic Resistance
Source: Cancers (Basel). 2020 Mar 13;12(3):675. doi: 10.3390/cancers12030675 (PMC7140110; doi:10.3390/cancers12030675)
Supplement: Supplementary file 1 [file cancers-12-00675-s001.zip › 717869-supplemental file-final/cancers-717869-supplementary file.pdf]

Supplemental table 1. *EGFR* variants in the *EGFR*-mutant cohort

EGFR variant	<30ng/ml 25(OH)D3	>30ng/ml 25(OH)D3
Exon 21 substitution, No (%)	7 (54)	5 (63)
Exon 19 deletion, No (%)	5 (38)	3 (37)
Compound	1 (8)	0 (0)
Total	13 (100)	8 (100)

Supplemental Table 2. *EGFR* genotypes for the models used in the current study

Model Name	*EGFR* genotype
H1975	Exon 20 T790M + Exon 21 L858R
H1975-OR	Exon 20 T790M + Exon 21 L858R
SH416	Exon 19 deletion
SH416-ER	Exon 19 deletion
PC9	Exon 19 deletion
PC9-ER	Exon 19 deletion
13T PDO	WT
VP2 PDO	Exon 19 deletion + Exon 20 T790M

Targeted sequencing of Exons 19-21 was performed according to the protocol described by Molina-Vila, et al. (JTO (2008) 3:1224-1235). For mutation detection, the resulting sequences were aligned against NCBI Reference Sequence NG_007726.3 using the ClustalW2 alignment tool. No mutations were detected other than those listed.

Supplemental Table 3. Primer Sequences

Gene	Forward, 5'-3'	Reverse, 5'-3'
EPCAM	GCTGGAATTGTTGTGCTGGTTA	AAGATGTCTTCGTCCCACGC
CLDN4	TCTCCTCTGTTCCGGGTAGG	CGTCCATCCACTCTGCACTT
MMP2	GGCTGTGTTCTTTGCAGGGAATGA	TGGGGAAGCCAGGATCCATTTTCT
GAPDH	CTCCTCTGACTTCAACAGCG	GCCAAATTCGTTGTCATACCAG
CYP24A1	GCACAAGAGCCTCAACACCAA	AGACTGTTTGCTGTCGTTTCCA
CAMP	TTCAAGAAGGACGGGCTGGTGAAG	GTCCTGGGTACAAGATTCCGCAAA

Supplemental Table 4. Statistical analysis of SH416-ER relative cell growth at day 7

	Vehicle	1,25(OH)2D3	Erlotinib	1,25(OH)2D3 +Erlotinib
Vehicle	NA	$p<0.05$	$p<0.01$	$p<0.0001$
1,25(OH)2D3	$p<0.05$	NA	$p=0.53$	$p<0.01$
Erlotinib	$p<0.01$	$p=0.53$	NA	$p<0.01$

Data were analyzed using one-way ANOVA with multiple comparisons.

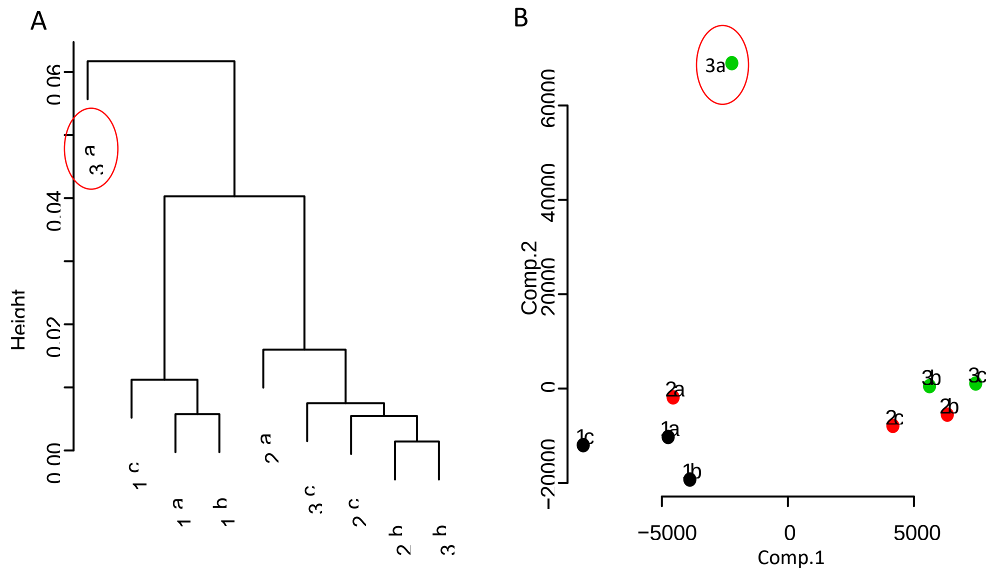

Supplemental Figure 1. Quality assessment analysis of the RNA sequencing results identified 3a sample as a deviation from the norm. Hierarchical clustering (A) and principal component analysis (B) revealed 3a as an atypical sample that strongly deviated in gene expression pattern from other samples in the same treatment group, as well as other samples in the same cell line. Therefore, sample 3a was excluded from further gene set enrichment analysis.

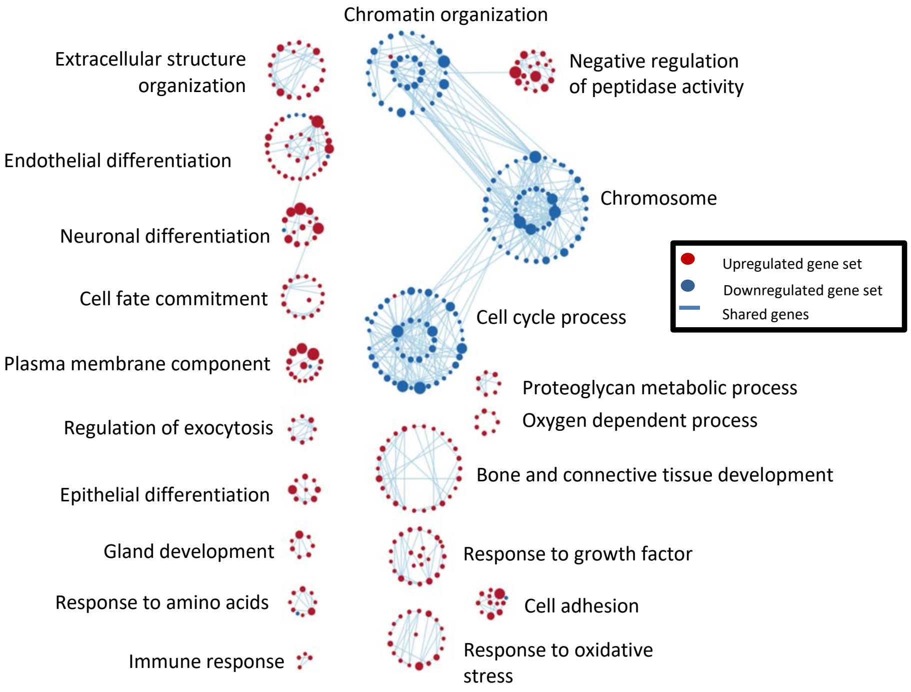

Supplemental Figure 2. Enrichment map of upregulated (red) and downregulated (blue) GO categories in H1975-OR cells treated with 1,25(OH)2D3. Significantly enriched gene sets are represented by colored dots. Gene sets are grouped into clusters (circles) based on the description of ontological categories to illustrate up- and down-regulated biological processes in H1975-OR cells treated with 1,25(OH)2D3.
